# 1135. The Effect of Penicillin Allergy Labels on Antibiotic Prescribing for Children Diagnosed with Upper Respiratory Tract Infections in Two Primary Care Networks

**DOI:** 10.1093/ofid/ofab466.1328

**Published:** 2021-12-04

**Authors:** Torsten Joerger, Margaret Taylor, Debra Palazzi, Jeffrey Gerber

**Affiliations:** 1 Lucile Packard Children’s Hospital/ Stanford University, Palo Alto, California; 2 Texas Children’s Hospital/ Baylor University, Houston, Texas; 3 Texas Children’s Hospital, Houston, TX; 4 Children’s Hospital of Philadelphia, Philadelphia, Pennsylvania

## Abstract

**Background:**

In pediatric inpatient settings, unconfirmed penicillin allergy labels (PALs) are associated with increased broad-spectrum antibiotic use, costs, and adverse events. However, 90% of antibiotics are prescribed in the outpatient setting and 70% of these antibiotics are given for upper respiratory tract infections (URTI.) Little is known about the effect of PALs on antibiotic prescribing in the pediatric outpatient population.

**Methods:**

A retrospective birth cohort was created of children born between January 1^st^ 2010 and June 30^th^ 2020 and seen at one of 91 Texas Children’s Pediatrics or Children’s Hospital of Philadelphia primary care clinics. Children with an ICD10 code for an URTI and an antibiotic prescription were stratified into those with or without a penicillin allergy label at the time of the infection. Rates of second-line and broad-spectrum antibiotic use were compared.

**Results:**

The birth cohort included 334,465 children followed for 1.2 million person-years. An antibiotic was prescribed for 696,782 URTIs and the most common diagnosis was acute otitis media. Children with PALs were significantly more likely to receive second-line antibiotics (OR 35.0, 95% CI 33.9-36.1) and broad-spectrum antibiotics (OR 23.9, 95% CI 23.2-24.8.) Children with PALs received more third generation cephalosporins (60% vs. 15%) and more macrolide antibiotics (25% vs. 3%) than those without a PAL. Overall, 18,015 children (5.4%) acquired a PAL during the study period, which accounted for 23% of all second-line antibiotic prescriptions and 17% of all broad-spectrum antibiotic use for URTIs.

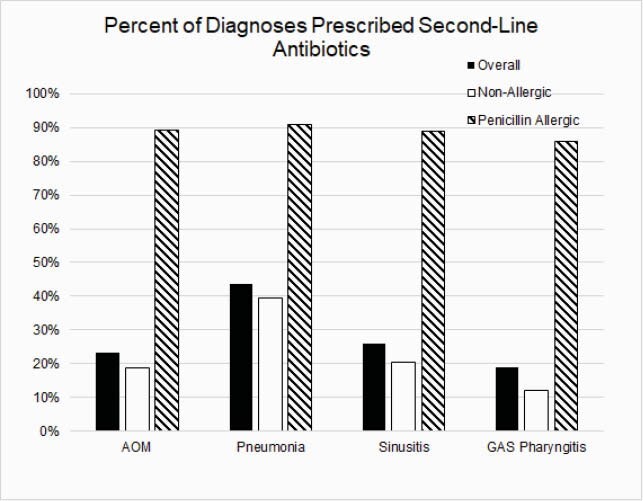

Multivariable logistic regression for receipt of second-line antibiotics for upper respiratory tract infections

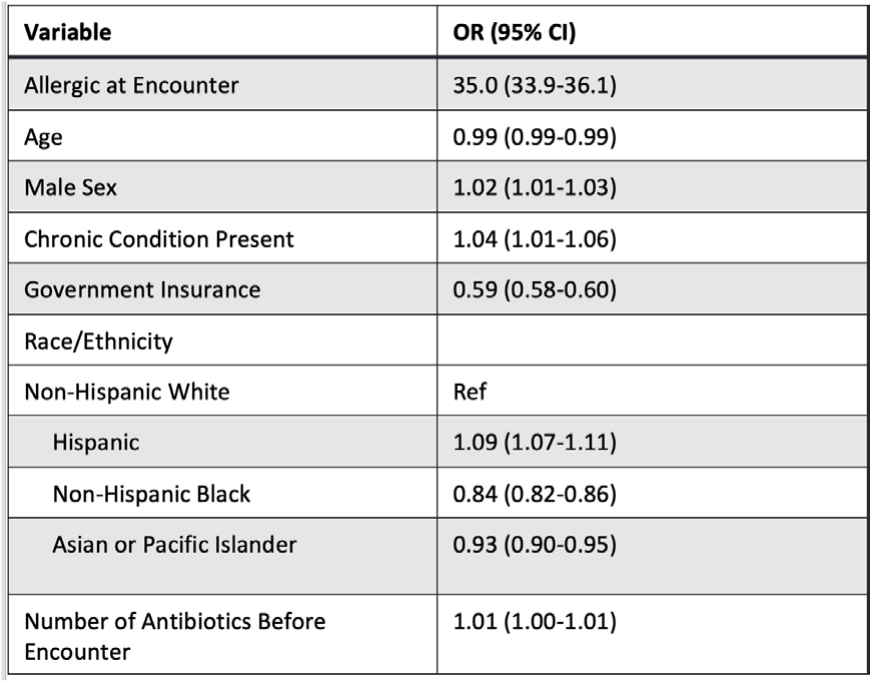

**Conclusion:**

PALs are common and account for a substantial proportion of second-line and broad-spectrum antibiotic use in pediatric outpatients treated for URTIs. Efforts to de-label children with PALs are likely to increase first-line antibiotic use and decrease broad-spectrum antibiotic use for URTIs, the most common indication for antibiotic prescribing to children.

**Disclosures:**

**Debra Palazzi, MD, MEd**, **AAP** (Other Financial or Material Support, PREP ID Editorial Board, PREP ID Course)**AHRQ** (Research Grant or Support)**Elsevier** (Other Financial or Material Support, Royalties for writing and editing chapters)**JAMA Pediatrics** (Board Member)

